# Screening and Engineering of Hetero-Bivalent Nanobody Targeting Interleukin-33 with Enhanced Binding Stability

**DOI:** 10.3390/biom16070936

**Published:** 2026-06-23

**Authors:** Yingxin Zhou, Leilei Shi, Weichen Wang

**Affiliations:** 1 School of Basic Medical Sciences, Wannan Medical University, Wuhu 241002, China; 2 Division of Life Sciences and Medicine, University of Science and Technology of China, Hefei 230001, China; sleil@ustc.edu.cn (L.S.); wangweichen@mail.ustc.edu.cn (W.W.)

**Keywords:** Interleukin-33, nanobody, phage display, hetero-bivalent engineering, binding stability

## Abstract

Interleukin-33 (IL-33) is an IL-1 family cytokine that functions as an alarmin and contributes to inflammatory responses, immune regulation, and tumor-associated processes through the IL-33/ST2 signaling axis. In this study, IL-33-specific nanobodies were isolated from a synthetic phage display library and further engineered into bivalent tandem formats to improve their binding performance. Five representative monovalent nanobodies showed concentration-dependent binding to IL-33, with SPR-derived *K*_D_ values ranging from 3.6 × 10^−8^ to 2.81 × 10^−7^ M. Among the engineered bivalent constructs, Nb1–Nb2 exhibited the strongest apparent binding affinity, mainly due to a markedly reduced dissociation rate. Competitive SPR analysis indicated that Nb1 and Nb2 show largely compatible binding to IL-33, consistent with distinct or minimally overlapping binding regions, supporting their selection as a hetero-bivalent pair. In a preliminary wound-healing assay using HT-29 colorectal cancer cells, Nb1–Nb2 attenuated IL-33-induced wound closure under low-serum conditions. These results indicate that hetero-bivalent engineering can enhance the apparent binding affinity of IL-33-targeting nanobodies and provide a useful molecular tool for further investigation of IL-33-associated biological responses.

## 1. Introduction

IL-33 is a member of the IL-1 cytokine family [1] and is abundantly expressed in the nuclei of endothelial cells, epithelial cells, and fibroblastic stromal cells in various tissues. It contains an N-terminal nuclear domain, a central protease sensor domain, and a C-terminal IL-1-like cytokine domain [2]. Upon cellular injury or necrotic cell death, IL-33 is passively released into the extracellular space, where it is cleaved by allergens or inflammatory proteases to generate mature IL-33 with increased biological activity [3,4]. The biological functions of IL-33 are mainly mediated through its interaction with the receptor ST2 (IL1RL1), which is highly expressed on group 2 innate lymphoid cells (ILC2s), mast cells, and regulatory T cells (Tregs) and is also detectable on multiple other immune cell types [5]. Binding of IL-33 to the membrane-bound isoform ST2L promotes recruitment of the IL-1 receptor accessory protein (IL-1RAcP), leading to the formation of a functional signaling complex and activation of the MyD88–IRAK–TRAF6 signaling axis [6]. This cascade activates downstream pathways such as NF-κB and MAPK, resulting in the induction of inflammatory mediators, tissue repair factors, and immune regulatory molecules [7]. Importantly, the biological outcomes of IL-33/ST2 signaling are highly context-dependent and can be either protective or pathogenic, depending on the cellular composition and the disease microenvironment [2,5,8].

In type 2 immune-driven diseases such as allergic disorders and asthma, IL-33 acts as an upstream cytokine that directly activates ILC2s, mast cells, and eosinophils [9,10]. This activation induces rapid production of IL-5 and IL-13 [11,12], thereby amplifying type 2 immune responses and promoting airway hyperresponsiveness, mucus hypersecretion, and chronic inflammation. Elevated IL-33 levels have been associated with asthma severity and exacerbation-prone phenotypes, supporting the involvement of IL-33 signaling in disease progression [13]. Consistent with these observations, neutralization of IL-33 in preclinical models markedly attenuates allergic inflammation [14]. Furthermore, clinical studies using monoclonal antibodies targeting IL-33 have demonstrated reduced eosinophil counts, improved disease control, and decreased rates of acute exacerbations, supporting the therapeutic relevance of IL-33 blockade in allergic diseases [15,16].

Beyond allergic inflammation, IL-33 has also emerged as an important regulator of tumor biology. Elevated IL-33 expression has been observed in several solid tumors, including colorectal, lung, and breast cancers [17,18,19]. Through ST2-dependent signaling, IL-33 promotes tumor cell survival and proliferation and contributes to remodeling of the tumor microenvironment by inducing inflammatory mediators and recruiting tumor-supportive immune cell populations [20]. Although IL-33 may enhance anti-tumor immunity under certain conditions, accumulating evidence indicates that sustained or dysregulated IL-33 signaling frequently facilitates tumor progression and immune evasion [21,22]. Notably, inhibition of the IL-33/ST2 axis has been shown to restrain tumor growth and enhance the efficacy of cancer immunotherapy, highlighting the therapeutic potential of IL-33 targeting in oncology [23,24].

Given the broad involvement of IL-33 in allergic inflammation, chronic airway diseases, and cancer, there is strong interest in developing strategies for precise and effective neutralization of IL-33. Although monoclonal antibodies targeting IL-33 have shown encouraging clinical efficacy, full-length antibody formats may be influenced by delivery route, tissue accessibility, and epitope accessibility in certain diseases [25,26]. Therefore, nanobodies, derived from the variable domains of camelid heavy-chain antibodies, offer several advantageous properties, including small size, high structural stability, strong antigen-binding affinity, and a modular architecture [27]. These features enable efficient microbial production, improved tissue penetration, and flexible engineering into multivalent or multispecific formats, making nanobodies well-suited for targeting soluble cytokines such as IL-33 and for modulating cytokine-mediated immune responses [28,29].

In the present study, we used phage display to isolate IL-33-specific nanobodies and further engineered selected clones into bivalent tandem formats. Biophysical characterization identified Nb1–Nb2 as a hetero-bivalent nanobody with markedly enhanced apparent binding stability, mainly due to a reduced dissociation rate. Competitive SPR analysis suggested largely compatible binding of Nb1 and Nb2 to IL-33, supporting their selection for hetero-bivalent engineering. In a wound-healing assay using HT-29 colorectal cancer cells, Nb1–Nb2 attenuated IL-33-induced wound closure under low-serum conditions. Together, these findings indicate that hetero-bivalent engineering can improve the binding performance of IL-33-targeting nanobodies and provide a useful molecular tool for further investigation of IL-33-associated biological responses.

## 2. Materials and Methods

### 2.1. Expression, Purification, and Biotinylation of IL-33 Protein

The human mature IL-33 fragment (residues 109–270) corresponding to the biologically active C-terminal cytokine domain was cloned into the pET-28a expression vector with an N-terminal 6 × His tag. The recombinant plasmid was transformed into *Escherichia coli* (*E. coli*) BL21(DE3) competent cells. Transformed cells were cultured in Terrific Broth (TB) medium supplemented with 50 μg/mL kanamycin at 37 °C with shaking at 220 rpm. When the optical density at 600 nm (OD_600_) reached 0.8–1.0, protein expression was induced by the addition of isopropyl β-D-1-thiogalactopyranoside (IPTG) to a final concentration of 0.5 mM, and the incubation temperature was reduced to 16 °C for overnight expression. Cells were harvested by centrifugation and resuspended in lysis buffer (50 mM Tris-HCl, 150 mM NaCl, pH 8.0). Cell disruption was performed using a high-pressure homogenizer (Guangzhou Juneng Nano & Bio Technology Co., Ltd., Guangzhou, China), followed by high-speed centrifugation. The supernatant of cell lysate was subjected to Ni-NTA affinity chromatography, and the eluted protein was further purified by size-exclusion chromatography.

For biotinylation, EZ-Link NHS-LC-LC-Biotin (Thermo Fisher Scientific, Waltham, MA, USA) was dissolved in dimethyl sulfoxide (DMSO) to a final concentration of 10 mM. The biotin reagent was added to purified IL-33 protein at a molar ratio of 1:10 (IL-33: biotin) and incubated on ice for 2 h under mild reaction conditions to minimize potential interference with antigenic epitopes. Unreacted free biotin was removed by buffer exchange using centrifugal ultrafiltration.

### 2.2. Construction and Amplification of the Nanobody Phage Display Library

A phage-displayed nanobody library was constructed using the pCANTAB5E phagemid vector. The library was designed by randomizing a 10-residue segment corresponding to amino acid positions 106–115 in the CDR3 region using NNK codons, while CDR1, CDR2, and the framework regions were kept constant. Briefly, the entire pCANTAB5E-based phagemid was amplified using degenerate primers containing NNK codons and flanking BsaI sites, thereby generating full-length linearized plasmids carrying randomized CDR3 sequences. The PCR products were digested with BsaI to remove the BsaI recognition sites and generate compatible ends for seamless circularization. The purified products were self-ligated using T4 DNA ligase and transformed into electrocompetent *E. coli* TG1 cells by electroporation. After recovery in SOC medium at 37 °C for 1 h, an aliquot of the transformed TG1 culture was serially diluted and plated on ampicillin-containing agar plates to estimate the number of independent transformants. This yielded a phagemid library of approximately 1 × 10^9^ independent transformants. The remaining transformed cells were cultured in 2 × YT medium supplemented with ampicillin and glucose at 37 °C and 220 rpm until the OD_600_ reached approximately 1.5, harvested, and stored as glycerol stocks at −80 °C.

Phage particles displaying the nanobody library were rescued using M13KO7 helper phage. The TG1 library stock was inoculated into 2 × YT medium containing 100 μg/mL ampicillin and 1% glucose and cultured at 37 °C and 220 rpm until the OD_600_ reached approximately 0.5. The cells were infected with M13KO7 helper phage at an MOI of approximately 20, incubated at 37 °C for 1 h without shaking, and then incubated for an additional 1 h with shaking. The infected cells were collected and cultured overnight at 26 °C and 220 rpm in 2 × YT medium supplemented with 100 μg/mL ampicillin and 25 μg/mL kanamycin. After overnight culture, the culture supernatant containing phage particles was collected by centrifugation and precipitated twice with PEG/NaCl at a 1:5 volume ratio. The final phage pellet was resuspended in PBST to obtain a freshly prepared phage-displayed nanobody library. Phage titers were determined by infecting *E. coli* TG1 cells, followed by serial dilution and plating on ampicillin-containing agar plates.

### 2.3. Phage Display-Based Selection of IL-33-Specific Nanobodies

Three rounds of phage display panning were performed against biotinylated IL-33. In the first round, 20 μL of streptavidin-coated magnetic beads (Thermo Fisher Scientific, Waltham, MA, USA) were incubated with 20 μg of biotinylated IL-33 at 4 °C for 1 h with gentle rotation. After washing with PBST (PBS containing 0.1% Tween-20), the antigen-coated beads were blocked with 300 μL of PBS containing 1% bovine serum albumin (BSA) for 1 h. The phage-displayed nanobody library, pre-blocked with PBS containing 1% BSA, was added at an input of approximately 1 × 10^13^ transducing units (TU) and incubated at 4 °C for 2 h. Unbound phages were removed by washing the beads 15 times with PBST, and bound phages were eluted with 200 μL of glycine buffer (pH 3.5), followed by immediate neutralization with Tris buffer. A portion of the eluate was used for phage titer determination, and the remainder was used to infect *E. coli* TG1 cells for amplification as the input for the next round.

To reduce enrichment of streptavidin- or bead-binding clones, NeutrAvidin-coated magnetic beads (Thermo Fisher Scientific, Waltham, MA, USA) were used in the second round following the same procedure. After amplification of the second-round output phages, a third round of selection was performed using streptavidin-coated magnetic beads. After the third round, eluted phages were used to infect *E. coli* TG1 cells, and individual colonies were picked and submitted for Sanger sequencing by Gene Universal. Sequencing was performed using primers covering the nanobody-coding region to identify enriched candidate nanobody sequences.

### 2.4. Construction, Expression, and Purification of Monovalent and Bivalent Nanobodies

Nanobody sequences obtained from phage display selection were cloned into the pET-28a vector with an N-terminal 6 × His-SUMO tag and a C-terminal FLAG tag. The resulting constructs were transformed into *E. coli* BL21(DE3) cells and plated on LB agar plates containing 50 μg/mL kanamycin. Single colonies were inoculated into 6 mL LB medium supplemented with kanamycin and cultured at 37 °C with shaking for 4–6 h, followed by transfer into 600 mL TB medium supplemented with kanamycin. When the OD_600_ reached 0.8–1.2, protein expression was induced by adding IPTG to a final concentration of 0.5 mM, and cultures were incubated at 16 °C overnight. Cells were harvested by centrifugation at 12,000× *g* for 30 min, resuspended in 40 mL TBS buffer, and lysed using a high-pressure homogenizer. The lysate was clarified by centrifugation at 12,000× *g* for 40 min, and the supernatant was subjected to Ni-NTA affinity chromatography. Proteins eluted from the Ni-NTA column were dialyzed against PBS for buffer exchange and subsequently purified using anti-FLAG affinity resin to further purify the nanobodies.

For bivalent tandem constructs, selected nanobody sequences were genetically fused in the indicated N-to-C-terminal order using a flexible (GGGGS)_3_ linker. The resulting Nb1–Nb1, Nb1–Nb2, and Nb1–Nb5 constructs were cloned into the same expression vector, and all final expression constructs, including the (GGGGS)_3_ linker regions, were verified by DNA sequencing. These bivalent nanobodies were then purified using the same procedure as described for monovalent nanobodies.

### 2.5. Enzyme-Linked Immunosorbent Assay (ELISA)

Recombinant IL-33 was diluted in PBS to 1 μg/mL and coated onto NUNC MaxiSorp plates (Thermo Fisher Scientific, Waltham, MA, USA) at 100 μL/well overnight at 4 °C. Plates were blocked with PBS containing 1% BSA (200 μL/well) for 2 h at room temperature. After washing three times with PBST (PBS containing 0.05% Tween-20), serially diluted nanobodies were tested at concentrations of 20, 10, 5, 2.5, 1, 0.5, 0.1, and 0.01 μg/mL (100 μL/well) and incubated at room temperature for 1–2 h. Plates were washed six times with PBST and incubated with HRP-conjugated anti-DDDDK (FLAG) antibody (ABclonal Technology, Wuhan, China) for 1–2 h at room temperature. After washing, the TMB substrate was added for color development, and the reaction was stopped with 1 M HCl. Absorbance was measured at 450 nm using a microplate reader, and data were analyzed with GraphPad Prism 8.

To assess nanobody-mediated inhibition of IL-33–ST2 binding, IL-33 was coated onto ELISA plates as described above. Recombinant ST2-Fc (Acro Biosystems, Beijing, China) at 1 μg/mL was pre-incubated with serially diluted monovalent or bivalent constructs and added to the plates for co-incubation at room temperature. After washing with PBST, bound ST2-Fc was detected using an HRP-conjugated anti-Fc antibody. TMB substrate was used for color development, and absorbance at 450 nm was recorded to generate inhibition curves.

Binding curves and EC_50_ values were obtained by nonlinear regression using a four-parameter logistic model in GraphPad Prism 8.

### 2.6. Surface Plasmon Resonance (SPR) Analysis

SPR measurements were performed using a Biacore T200 instrument (GE Healthcare, Uppsala, Sweden) to characterize the binding kinetics between nanobodies and IL-33. Recombinant IL-33 was immobilized on a CM5 sensor chip via standard amine coupling. Briefly, both ligand and reference flow channels were activated with an EDC/NHS mixture, and IL-33 diluted in 10 mM sodium acetate buffer (pH 4.0) was injected at a flow rate of 5 μL/min to achieve an immobilization level of approximately 100 response units (RU). The remaining activated groups were blocked with ethanolamine. All experiments were conducted using PBS containing 0.05% Tween-20 as the running buffer. For kinetic analysis, Nb1, Nb2, Nb3, and Nb5 were injected at serially diluted concentrations up to 1000 nM, whereas Nb4 was further analyzed up to 3000 nM because of its relatively low response at the initial tested concentrations. For each concentration, association and dissociation were monitored for 90 s and 180 s, respectively, at a flow rate of 30 μL/min. The sensor surface was regenerated with glycine-HCl (pH 2.5) between injection cycles. Sensorgrams were reference-subtracted and fitted using a 1:1 Langmuir binding model. For bivalent tandem nanobodies, the fitted kinetic parameters were interpreted as apparent kinetic values.

For competitive SPR analysis, a sequential injection protocol was used. Nb2 or Nb5 was first injected over the IL-33-immobilized surface, followed by injection of either the same nanobody alone or a mixture containing the same nanobody and Nb1. Thus, Nb2/Nb2 + Nb1 and Nb5/Nb5 + Nb1 competition assays were performed. Nb2, Nb5, and Nb1 were used at 2500, 5000, and 2000 nM, respectively. All injections were performed at a flow rate of 30 μL/min for 180 s, followed by a 120 s dissociation phase. Buffer injection followed by Nb1 injection was used as a control.

### 2.7. Wound-Healing Assay

HT-29 colorectal cancer cells were obtained from the American Type Culture Collection (ATCC, Manassas, VA, USA; catalog number: HTB-38). The cells were cultured in DMEM supplemented with 10% fetal bovine serum (FBS), 100 U/mL penicillin, and 100 μg/mL streptomycin and allowed to reach full confluence in six-well plates. A linear scratch was created using a 10 μL pipette tip, and detached cells were removed by gently washing twice with PBS. Cells were then cultured under three conditions: (i) DMEM containing 1% FBS, (ii) DMEM containing 1% FBS supplemented with IL-33 (50 ng/mL), and (iii) DMEM containing 1% FBS supplemented with IL-33 (50 ng/mL) and Nb1–Nb2 (10 μg/mL, approximately 234 nM). Plates were incubated at 37 °C in a humidified atmosphere with 5% CO_2_. Images of the same wound areas were acquired at 0 and 24 h using a cell imaging system. Wound closure was quantified using ImageJ software (version 1.54g) and calculated as follows: Wound closure (%) = [(wound area at 0 h − wound area at 24 h)/wound area at 0 h] × 100%. Results were compared between treatment groups and their corresponding controls. The wound-healing assay was independently repeated three times. In each independent experiment, two replicate wells were analyzed for each condition, and the mean value of the two replicate wells was used as one biological replicate for statistical analysis.

### 2.8. Statistical Analysis

Data are presented as mean ± SD unless otherwise indicated. Statistical analyses were performed using GraphPad Prism 8. Comparisons among multiple groups were conducted using one-way ANOVA followed by Tukey’s multiple comparisons test. A value of *p* < 0.05 was considered statistically significant.

## 3. Results

### 3.1. Nanobody Library Screening Against IL-33

**Figure 1:**
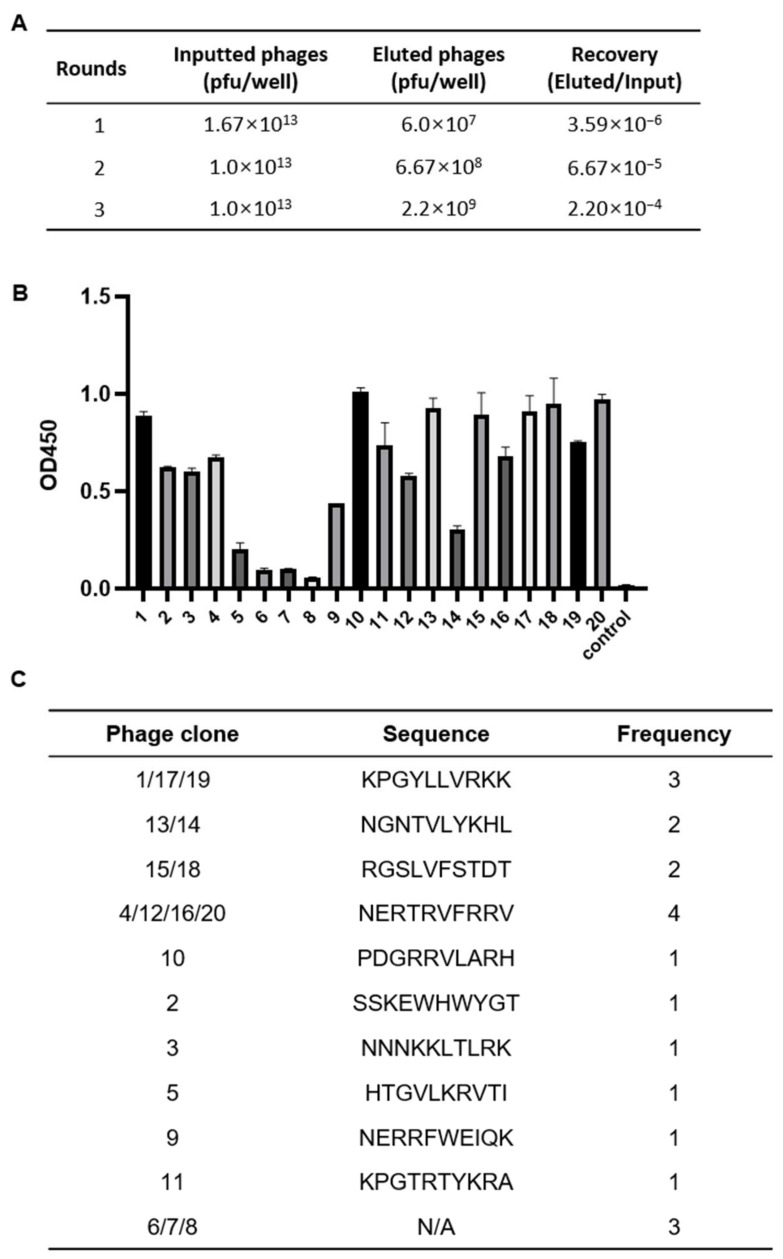
Selection of IL-33-targeting nanobodies by phage display. (**A**) Phage recovery after each round of biopanning. (**B**) Evaluation of phage binding to IL-33 by phage ELISA. A total of 20 individual phage clones were analyzed, with PBS used as the negative control. (**C**) Sequence and frequency analysis of individual phage clones picked after the final round of biopanning.

To generate a homogeneous antigen for nanobody selection, a recombinant construct corresponding to the mature human IL-33 cytokine domain (residues 109–270) was expressed in *E. coli* BL21(DE3). SDS–PAGE analysis showed that IL-33 was efficiently expressed in the soluble fraction. The protein was subsequently purified by Ni-NTA affinity chromatography followed by size-exclusion chromatography, yielding IL-33 with high purity (Figure S1A). Purified IL-33 was biotinylated and immobilized on streptavidin-coated magnetic beads for phage display selection. Screening was performed using a synthetic M13 phage-displayed nanobody library previously constructed in our laboratory, which was designed based on a conserved nanobody framework with randomized 10-amino-acid CDR3 sequences and a diversity of approximately 10^9^. Three consecutive rounds of biopanning were carried out, with approximately 1 × 10^13^ phage particles applied in each round. A progressive enrichment of eluted phages was observed, with an approximately 61-fold increase in the third round compared to that of the first round, indicating effective enrichment of IL-33-specific binders (Figure 1A).

Following phage display selection, twenty clones were randomly selected. Binding of individual clones to IL-33 was assessed by phage ELISA, in which IL-33-bound phages were detected using an HRP-conjugated anti-M13 antibody and quantified by absorbance at 450 nm. Most clones showed strong binding signals, with A_450_ values more than tenfold higher than those of negative controls, whereas clones 5–8 exhibited weak binding (Figure 1B). DNA sequencing of selected clones further revealed an uneven distribution of sequence frequencies, with several families dominating the pool, suggesting preferential selection during panning (Figure 1C).

### 3.2. Purification and Affinity Characterization of IL-33-Specific Nanobodies

**Figure 2:**
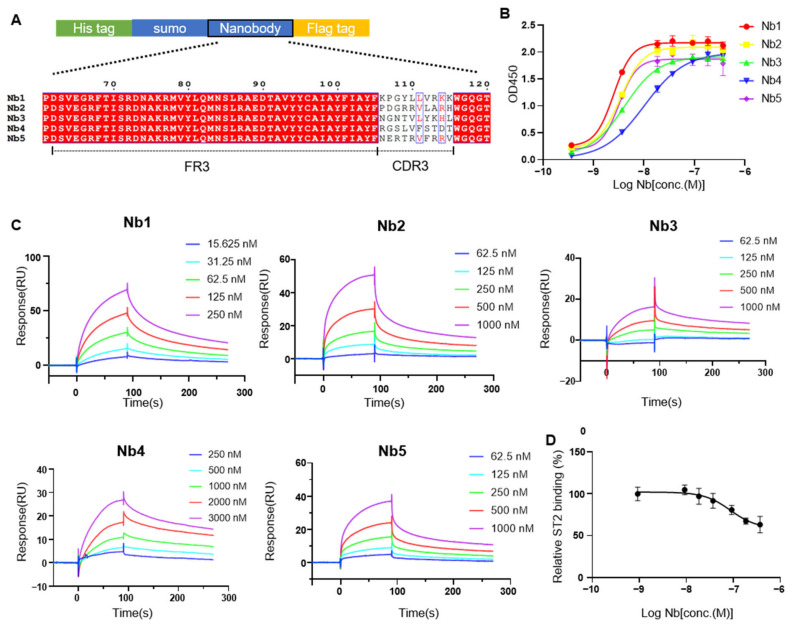
Expression and functional characterization of IL-33-targeting nanobodies. (**A**) Schematic illustration of nanobody expression constructs showing the CDR3 amino acid sequences of Nb1 to Nb5. (**B**) Binding of nanobodies to IL-33 measured by indirect ELISA. FLAG-tagged nanobodies were detected using an HRP-conjugated anti-FLAG antibody. (**C**) SPR analysis of nanobody binding to IL-33. IL-33 was immobilized on a CM5 sensor chip, and serially diluted nanobodies were injected. Sensorgrams were reference-subtracted and fitted using a 1:1 binding model. (**D**) Competitive ELISA evaluating Nb1 interference with IL-33–ST2-Fc interaction. IL-33-coated plates were incubated with ST2-Fc (1 μg/mL) pre-incubated with serially diluted Nb1, and bound ST2-Fc was detected using an HRP-conjugated anti-Fc antibody.

Based on sequence frequency analysis and phage ELISA results following phage display selection, five representative nanobody clones (termed Nb1, Nb2, Nb3, Nb4, and Nb5) were selected for recombinant expression (Figure 2A). To improve soluble expression of nanobodies, a SUMO-tag fusion strategy was applied, as previously reported [30]. The fusion proteins were then purified by a tandem Ni-NTA and anti-FLAG affinity chromatography, followed by size-exclusion chromatography to achieve high purity as determined by Coomassie staining. Using this strategy, all five IL-33-specific nanobodies were successfully obtained in soluble form for subsequent characterization (Figure S2A,B).

The binding affinity of purified nanobodies toward IL-33 was first evaluated by ELISA. All five nanobodies exhibited concentration-dependent binding to IL-33, with EC_50_ values of 2.52 nM (Nb1), 3.46 nM (Nb2), 4.16 nM (Nb3), 10.24 nM (Nb4), and 3.23 nM (Nb5), respectively (Figure 2B). SPR analysis further confirmed IL-33 binding by the selected nanobodies. Nb1 displayed the fastest association rate (*k*_a_ = 4.24 × 10^5^ M^−1^·s^−1^), and despite a relatively rapid dissociation rate (*k*_d_ = 1.52 × 10^−2^ s^−1^), it showed the highest apparent binding affinity, with an equilibrium dissociation constant (*K*_D_) of 3.6 × 10^−8^ M. The remaining nanobodies (Nb2 to Nb5) exhibited moderate binding affinities, with *K*_D_ values ranging from 1.21 × 10^−7^ to 2.81 × 10^−7^ M (Figure 2C and Table 1). These results indicate that all selected monovalent nanobodies can bind IL-33, with Nb1 exhibiting the highest binding affinity.

To test if Nb1 can inhibit the interaction between IL-33 and its receptor ST2, we performed a competitive ELISA in which IL-33-coated plates were incubated with ST2-Fc premixed with serially diluted Nb1. Nb1 showed only limited inhibition of IL-33–ST2 binding (Figure 2D). To explore a possible structural basis for this weak inhibitory effect, AlphaFold3-based modeling of the IL-33–Nb1 complex was performed and compared with the reported IL-33–ST2 complex structure (Figure S2C). IL-33 interacts with ST2 through two major interfaces, with Site 1 serving as the primary receptor-binding interface and Site 2 contributing to complex stabilization [31]. The predicted IL-33–Nb1 model suggested that Nb1 may bind near Site 2 rather than directly occupying the primary ST2-binding interface. This possible binding mode may partly explain the limited blockade of IL-33–ST2 binding observed in the competitive ELISA and appears in line with a previous report [32]. In addition, the relatively rapid dissociation of Nb1 may also contribute to its limited inhibitory effect.

**Table 1:** Binding kinetics of nanobodies to IL-33 determined by SPR. *K*_D_, equilibrium dissociation constant; *k*_a_, association rate constant; *k*_d_, dissociation rate constant.

Nanobody	*k*_a_ (M^−1^·s^−1^)	*k*_d_ (s^−1^)	*K*_D_ (M)
Nb1	4.24 × 10^5^	1.52 × 10^−2^	3.60 × 10^−8^
Nb2	4.14 × 10^4^	6.28 × 10^−3^	1.52 × 10^−7^
Nb3	3.69 × 10^4^	4.45 × 10^−3^	1.21 × 10^−7^
Nb4	2.89 × 10^4^	5.40 × 10^−3^	1.87 × 10^−7^
Nb5	1.90 × 10^4^	5.34 × 10^−3^	2.81 × 10^−7^

### 3.3. Purification and Affinity Characterization of Bivalent Nanobodies

To explore whether bivalent engineering could improve IL-33 binding, Nb1, Nb2, and Nb5 were selected for tandem nanobody construction. Nb1 showed the highest apparent binding affinity among the tested clones, Nb2 represented a moderate-affinity binder, and Nb5 was the most frequently enriched sequence after phage display panning. Bivalent constructs were generated by linking Nb1–Nb1, Nb1–Nb2, or Nb1–Nb5 using a flexible (GGGGS)_3_ linker to provide conformational flexibility between VHH domains [33,34]. Bivalent nanobody constructs were designed and expressed as SUMO-tag fusion proteins and purified by sequential His-tag and FLAG-tag affinity chromatography followed by size-exclusion chromatography. As a representative example, SDS–PAGE analysis and size-exclusion chromatography profiles of the Nb1–Nb2 bivalent construct are shown in Figure S3A,B.

**Figure 3:**
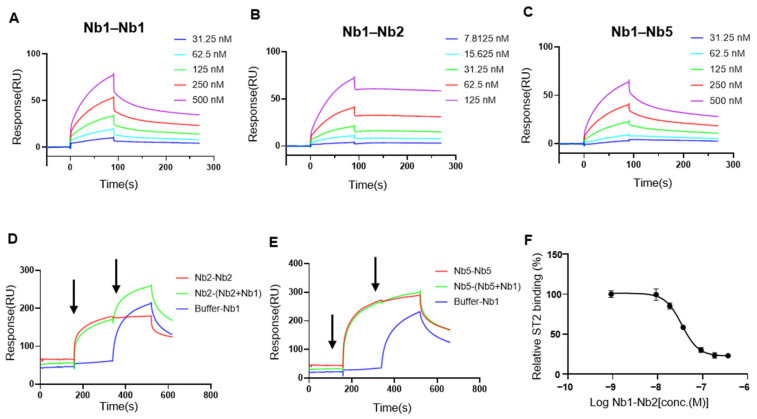
Biophysical characterization of IL-33-targeting bivalent nanobodies. (**A**–**C**) SPR analysis of bivalent nanobody binding to IL-33. Nb1–Nb1 (**A**), Nb1–Nb2 (**B**), and Nb1–Nb5 (**C**). IL-33 was immobilized on a CM5 sensor chip, and serially diluted bivalent nanobodies were injected. Sensorgrams were analyzed using Biacore T200 Evaluation Software (version 3.0). (**D**) Competitive SPR analysis of Nb1 and Nb2 binding to IL-33. Nb2 was injected to reach a binding plateau, followed by injection of Nb2 alone or a Nb2/Nb1 mixture. Buffer followed by Nb1 injection served as control. (**E**) Competitive SPR analysis of Nb1 and Nb5 binding to IL-33. Nb5 was injected to reach a binding plateau, followed by injection of Nb5 alone or a Nb5/Nb1 mixture. Buffer followed by Nb1 injection served as control. (**F**) Competitive ELISA evaluating Nb1–Nb2 inhibition of IL-33–ST2 interaction. IL-33-coated plates were incubated with ST2-Fc pre-mixed with serially diluted hetero-bivalent Nb1–Nb2, and bound ST2-Fc was detected using an HRP-conjugated anti-Fc antibody. The black arrows in subfigures D and E indicate the two injection time points during the sequential SPR injection assay.

The binding kinetics of the three bivalent nanobody formats to IL-33 were subsequently characterized by surface plasmon resonance (Figure 3A–C). Among the tested tandem nanobodies, Nb1–Nb2 showed the most pronounced enhancement in apparent binding affinity. Kinetic analysis revealed that Nb1–Nb2 displayed a rapid association rate constant (*k*_a_ = 1.82 × 10^5^ M^−1^·s^−1^) together with a markedly reduced dissociation rate constant (*k*_d_ = 2.01 × 10^−4^ s^−1^), resulting in an apparent equilibrium dissociation constant (apparent *K*_D_) of 1.10 × 10^−9^ M (Table 2). Compared with the homo-bivalent Nb1–Nb1 construct, the hetero-bivalent Nb1–Nb2 format showed a more pronounced reduction in dissociation rate, suggesting that hetero-bivalent engineering was more effective than simple duplication of Nb1 in improving apparent binding stability.

To obtain preliminary information on binding-region compatibility among Nb1, Nb2, and Nb5, SPR competition assays were performed using a sequential injection strategy. In the Nb2/Nb1 assay, Nb2 pre-binding did not prevent additional Nb1 binding, as the Nb2/Nb1 mixture generated an additional response compared with Nb2 alone, indicating that Nb1 can still engage IL-33 after Nb2 binding (Figure 3D). In contrast, Nb1 showed only a limited additional response after Nb5 pre-binding, suggesting partial competition or steric hindrance between Nb1 and Nb5 (Figure 3E). Overall, these competition data suggest that Nb1 and Nb2 can bind IL-33 in a largely compatible manner, consistent with distinct or minimally overlapping binding regions, whereas Nb1 and Nb5 may be partially competitive or sterically constrained. This observation is broadly consistent with AlphaFold3-based modeling, which predicted spatially separated binding regions for Nb1 and Nb2 (Figure S2D). Consistently, competitive ELISA showed that Nb1–Nb2 reduced IL-33–ST2 binding to ~20% (Figure 3F), whereas monovalent Nb1 achieved ~60% under the tested conditions (Figure 2D), suggesting stronger blockade by the hetero-bivalent format.

### 3.4. Preliminary Evaluation of Nb1–Nb2 in an IL-33-Induced HT-29 Wound-Healing Assay

**Figure 4:**
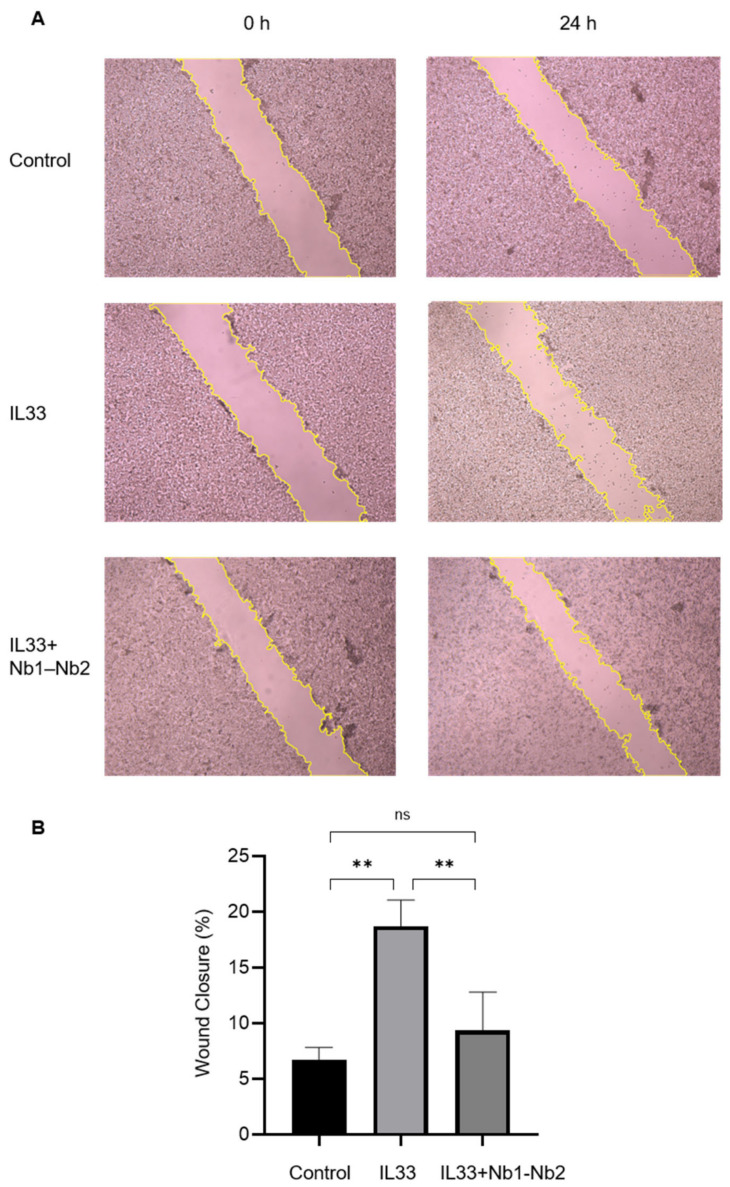
Hetero-bivalent nanobody Nb1–Nb2 attenuates IL-33-induced wound closure in HT-29 cells. (**A**) Representative images of HT-29 cell cultures at 0 h and 24 h after wounding. Cells were maintained in DMEM containing 1% FBS throughout the experiment. For IL-33 treatment, IL-33 (50 ng/mL) was added to the medium. For combined treatment, cells were incubated with IL-33 (50 ng/mL) together with the hetero-bivalent nanobody Nb1–Nb2 (10 μg/mL). The yellow lines mark the wound edges automatically identified using ImageJ for wound-closure quantification. (**B**) Quantification of wound closure at 24 h, expressed as the percentage of wound closure relative to 0 h. Data are presented as mean ± SD from three independent experiments. In each independent experiment, two replicate wells were analyzed for each condition, and the mean value of the two wells was used as one biological replicate for statistical analysis. Statistical significance was assessed by one-way ANOVA with Tukey’s multiple comparisons test, and all pairwise comparisons among the indicated groups are shown. ns, not significant; **, *p* < 0.01.

To evaluate the cell-based effect of the bivalent nanobody Nb1–Nb2 on IL-33–mediated cellular responses, a wound-healing assay was performed using HT-29 colorectal cancer cells. Representative wound images under the indicated treatment conditions are shown in Figure 4A. Under low-serum conditions (1% FBS), HT-29 cells exhibited limited basal wound closure, as minimal change in wound area was observed over the incubation period. In contrast, supplementation with exogenous IL-33 (50 ng/mL) under the same low-serum conditions resulted in a statistically significant increase in wound area closure relative to the control, indicating that IL-33 promoted wound closure in this assay, consistent with previous observations [35].

In the presence of both IL-33 and Nb1–Nb2 (10 μg/mL, approximately 234 nM), wound closure was significantly reduced compared with the group supplemented with exogenous IL-33. Notably, wound closure in the IL-33 + Nb1–Nb2 group was not significantly different from that in the control group, suggesting that Nb1–Nb2 attenuated IL-33-associated wound-closure response under the tested conditions. Quantitative analysis of wound area changes is summarized in Figure 4B. These results provide preliminary cell-based evidence that the Nb1–Nb2 can modulate an IL-33-associated wound-closure response.

## 4. Discussion

IL-33 is an inflammation-associated cytokine that signals mainly through the IL-33/ST2 axis and has been implicated in allergic inflammation and tumor-associated processes. Therapeutic modulation targeting the IL-33/ST2 axis therefore represents an attractive strategy for molecular intervention [36]. In this study, IL-33-specific monovalent nanobodies were obtained by phage display selection, and bivalent nanobodies were further engineered to evaluate whether a multivalent design could improve IL-33-binding performance.

Although Nb1 showed the highest apparent affinity among the monovalent nanobodies, its dissociation from IL-33 was relatively rapid, with a *k*_d_ value of 1.52 × 10^−2^ s^−1^ and an estimated dissociation half-life of approximately 46 s. By contrast, the Nb1–Nb2 construct displayed a markedly reduced apparent *k*_d_ of 2.01 × 10^−4^ s^−1^, corresponding to an apparent dissociation half-life of approximately 57 min. This approximately 75-fold reduction in the apparent dissociation rate indicates that the enhanced SPR binding profile of Nb1–Nb2 was mainly attributable to prolonged apparent target residence time. These findings suggest that hetero-bivalent engineering can improve the binding performance of IL-33-targeting nanobodies primarily by stabilizing target engagement.

Structurally, IL-33 interacts with its receptor ST2 through multiple binding interfaces [37]. In this study, competitive SPR analysis showed that Nb1 could generate an additional binding response after Nb2 had already bound to IL-33, which is consistent with distinct or minimally overlapping binding regions of Nb1 and Nb2. These data provide preliminary support for compatible binding behavior of Nb1 and Nb2 on IL-33 and may be associated with the improved apparent binding stability of the Nb1–Nb2 format.

At the cellular level, wound-healing assays using HT-29 colorectal cancer cells showed that exogenous IL-33 promoted wound closure under low-serum conditions, consistent with previous reports linking the IL-33/ST2 pathway to colorectal cancer invasion- and metastasis-related phenotypes [38]. In contrast, co-treatment with Nb1–Nb2 reduced this IL-33-induced response, suggesting that Nb1–Nb2 can attenuate an IL-33-associated wound-closure response under the tested conditions.

Nevertheless, several limitations should be noted. First, although the (GGGGS)_3_ linker was selected as a flexible spacer commonly used in multivalent nanobody engineering, linker optimization was not performed in this study. Because linker length and flexibility may influence the spatial arrangement and avidity of bivalent binding, systematic evaluation of alternative linkers with different lengths or rigidities may help further optimize the binding properties of the Nb1–Nb2 construct.

Second, the functional validation of Nb1–Nb2 remains preliminary. Although the competitive ELISA showed that Nb1–Nb2 inhibited IL-33–ST2 binding more effectively than monovalent Nb1, this assay only measures the IL-33–ST2-Fc interaction and does not assess the IL-33/ST2/IL-1RAcP ternary signaling complex. Similarly, the HT-29 wound-healing assay showed that Nb1–Nb2 attenuated an IL-33-associated wound-closure response, but wound closure can be influenced by multiple cellular processes and does not directly measure ST2-dependent downstream signaling. Importantly, equimolar monovalent Nb1 and Nb2 controls were not included in the wound-healing assay. Therefore, the current data do not allow determination of whether the attenuation of IL-33-induced wound closure was format-dependent or reflected the activity of individual nanobody domains. Future studies incorporating monovalent controls, pathway-specific signaling assays, cytokine production assays, and ternary-complex formation assays will be needed to clarify the blocking mechanism and format-dependent functional activity of Nb1–Nb2.

Third, the structural basis of Nb1–Nb2 binding to IL-33 remains incompletely defined. Although AlphaFold3-based binary modeling suggested putative binding regions for Nb1 and Nb2, reliable modeling for IL-33 in complex with Nb1–Nb2 could not be obtained in this study. Therefore, the spatial arrangement of Nb1 and Nb2 on IL-33 remains unresolved, and whether the two nanobody domains within the Nb1–Nb2 construct can simultaneously engage their binding regions on the same IL-33 molecule remains to be determined. Further validation using site-directed mutagenesis, experimental epitope mapping, or high-resolution structural studies will be required to define the precise binding sites of Nb1–Nb2 on IL-33.

More broadly, the present results emphasize the importance of epitope selection in IL-33-targeting binder design. Systematic evaluation of additional nanobody combinations, guided by epitope binning and orthogonal functional assays, will be necessary to fully optimize hetero-bivalent design strategies for IL-33 targeting. IL-33 interacts with ST2 through spatially distributed interfaces, while binders targeting different regions may vary substantially in their ability to block receptor interaction or downstream activity. Computational epitope-guided protein design may help expand the accessible epitope space, but the resulting binders will still require experimental validation and affinity maturation using display-based platforms [39]. Integrating computational epitope targeting with phage display selection may therefore provide a useful strategy for developing IL-33 binders with improved binding and functional properties.

## 5. Conclusions

This study identified IL-33-specific nanobodies by phage display and showed that hetero-bivalent engineering can markedly enhance their apparent binding stability, mainly by reducing the dissociation rate. The Nb1–Nb2 tandem nanobody showed improved apparent target engagement, and competitive SPR analysis provided preliminary evidence for largely compatible binding behavior between Nb1 and Nb2 on IL-33. In addition, Nb1–Nb2 attenuated IL-33-induced wound closure in HT-29 cells, providing preliminary evidence of IL-33-associated cellular modulation under the tested conditions. These findings support hetero-bivalent engineering as a useful strategy for improving the apparent binding stability of IL-33-targeting nanobodies and provide a molecular tool for further investigation of IL-33-associated signaling and IL-33-targeting biologic design.

## Figures and Tables

**Table 2:** Apparent binding kinetics of bivalent nanobodies to immobilized IL-33 determined by SPR. *K*_D_, equilibrium dissociation constant; *k*_a_, association rate constant; *k*_d_, dissociation rate constant.

Nanobody	*k*_a_ (M^−1^·s^−1^)	*k*_d_ (s^−1^)	*K*_D_ (M)
Nb1–Nb1	8.07 × 10^4^	3.83 × 10^−3^	4.75 × 10^−8^
Nb1–Nb2	1.82 × 10^5^	2.01 × 10^−4^	1.10 × 10^−9^
Nb1–Nb5	4.02 × 10^4^	3.43 × 10^−3^	8.54 × 10^−8^

## Data Availability

The data presented in this study are available in this article and the Supplementary Materials. Further inquiries may be directed to the corresponding author.

## Supplementary Materials

The following supporting information can be downloaded at https://www.mdpi.com/article/10.3390/biom16070936/s1, Figure S1: Purification of recombinant human IL-33; Figure S2: Expression, purification, and computational structural modeling of monovalent IL-33-targeting nanobodies.; Figure S3: Purification of IL-33-targeting bivalent nanobodies.

## Abbreviations

The following abbreviations are used in this manuscript:

IL-33	Interleukin-33
IL-1	Interleukin-1
ST2	Suppression of tumorigenicity 2
IL1RL1	Interleukin-1 receptor-like 1
IL-1RAcP	IL-1 receptor accessory protein
ILC2s	Group 2 innate lymphoid cells
Tregs	Regulatory T cells
MyD88	Myeloid differentiation primary response 88
IRAK	Interleukin-1 receptor-associated kinase
TRAF6	Tumor necrosis factor receptor-associated factor 6
NF-κB	Nuclear factor kappa B
MAPK	Mitogen-activated protein kinase
Nb	Nanobody
ELISA	Enzyme-linked immunosorbent assay
SPR	Surface plasmon resonance
CDR3	Complementarity-determining region 3
SUMO	Small ubiquitin-like modifier
IPTG	Isopropyl β-D-1-thiogalactopyranoside
