# Supplementary material for: Screening and Engineering of Hetero-Bivalent Nanobody Targeting Interleukin-33 with Enhanced Binding Stability
Source: Biomolecules. 2026 Jun 23;16(7):936. doi: 10.3390/biom16070936 (PMC13406748; doi:10.3390/biom16070936)
Supplement: Supplementary file 1 [file biomolecules-16-00936-s001.zip › biomolecules-4346368-supplementary.pdf]

biomolecules

Supplementary Materials

Screening and Engineering of Hetero-bivalent Nanobody Targeting Interleukin-33 with Enhanced Binding Stability

Yingxin Zhou [1]*, Leilei Shi [2], Weichen Wang [2]

[1] School of Basic Medical Sciences, Wannan Medical University, Wuhu, Anhui 241002, China; yxzhou@mail.ustc.edu.cn
[2] Division of Life Sciences and Medicine, University of Science and Technology of China, Hefei, Anhui 230001, China; sleil@ustc.edu.cn; wangweichen@mail.ustc.edu.cn
* Correspondence: yxzhou@mail.ustc.edu.cn;

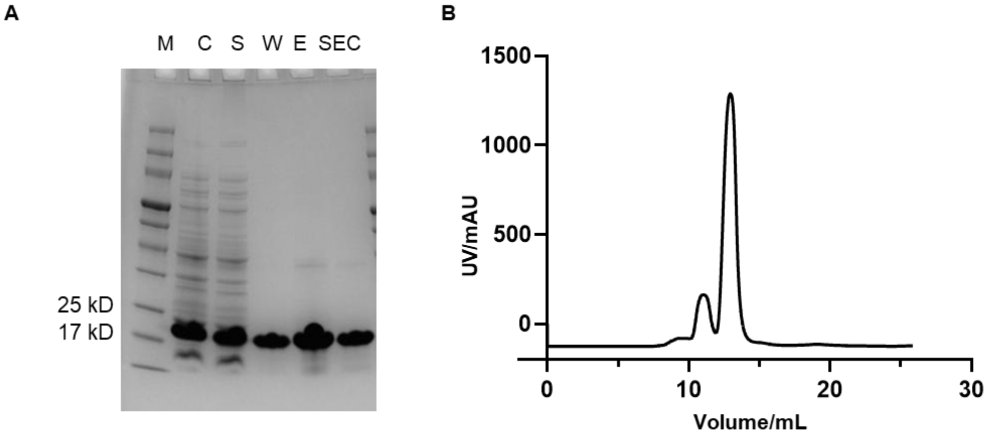

Figure S1. Purification of recombinant human IL-33. (A) SDS-PAGE analysis of IL-33 at different stages of purification. M, protein marker; C, whole-cell lysate; S, soluble fraction; W, wash fraction with 20 mM imidazole; E, elution fraction with 250 mM imidazole; SEC, sample after size-exclusion chromatography. (B) Size-exclusion chromatography (SEC) profile of purified IL-33.

Academic Editor:

Received:
Revised:
Accepted:
Published:

Copyright: © 2026 by the authors. Submitted for possible open access publication under the terms and conditions of the Creative Commons Attribution (CC BY) license.

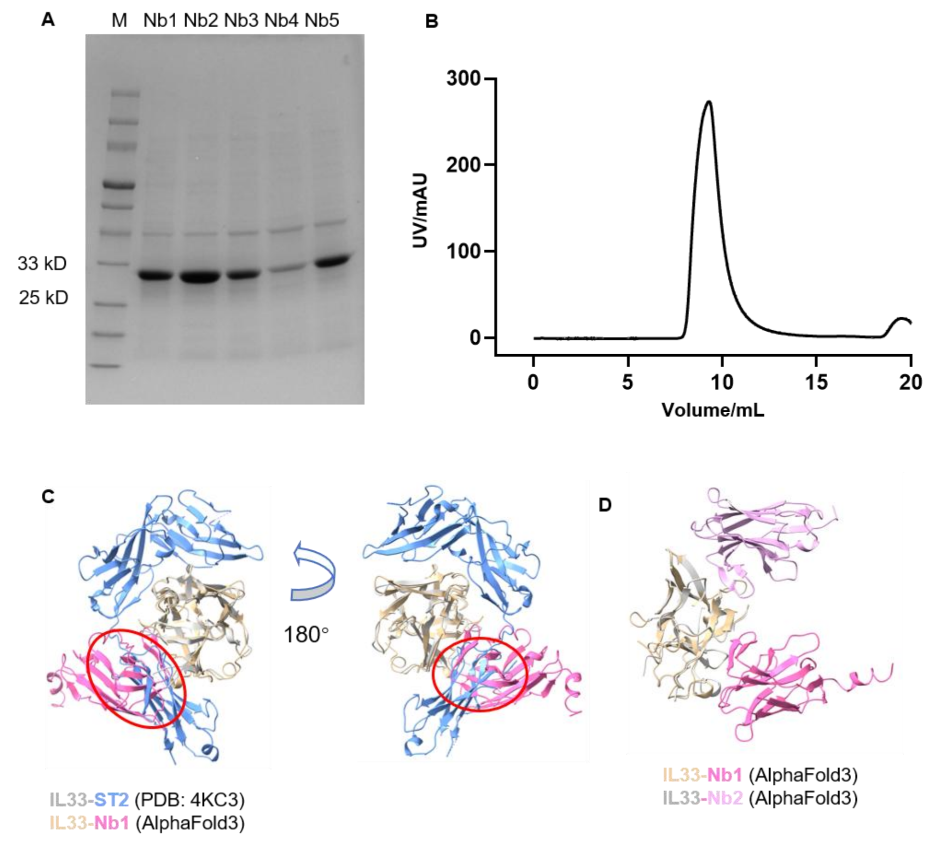

Figure S2. Expression, purification, and computational structural modeling of monovalent IL-33–targeting nanobodies. (A) SDS-PAGE analysis of purified SUMO-fusion nanobodies. M, protein marker; Nb1 to Nb5, nanobody clones 1 to 5. (B) Representative size-exclusion chromatography (SEC) profile of Nb1. (C) Structural modeling of the IL-33–Nb1 complex and comparison with the IL-33–ST2 complex. In the IL-33–ST2 complex (PDB ID: 4KC3), IL-33 is shown in gray and ST2 in blue. In the IL-33–Nb1 complex predicted by AlphaFold3, IL-33 is shown in yellow and Nb1 in pink. (D) Structural comparison of IL-33–Nb1 and IL-33–Nb2 complexes aligned on IL-33. In the AlphaFold3-predicted IL-33–Nb2 complex, IL-33 is shown in gray and Nb2 in magenta.

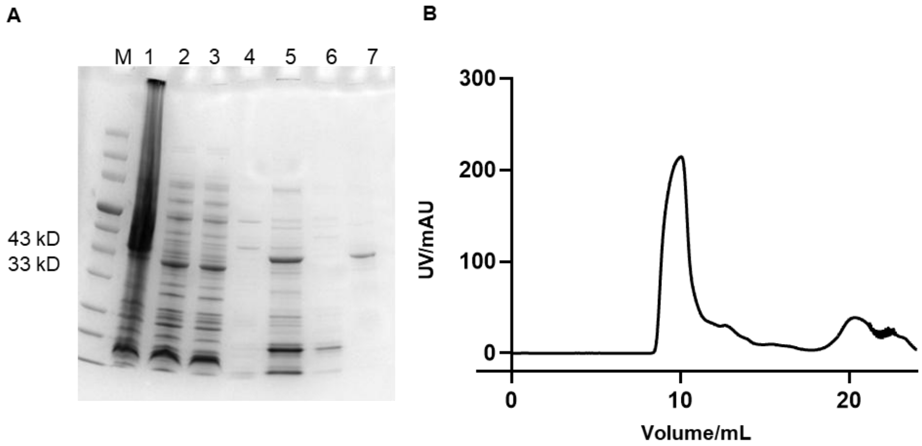

Figure S3. Purification of IL-33–targeting bivalent nanobodies. (A) SDS-PAGE analysis of Nb1–Nb2 bivalent construct during purification. M, protein marker; lane 1, whole-cell lysate; lane 2, soluble fraction; lane 3, flow-through after Ni-NTA loading; lane 4, wash fraction containing 20 mM imidazole; lane 5, elution fraction containing 250 mM imidazole; lane 6, flow-through after anti-FLAG affinity chromatography; lane 7, elution fraction with FLAG peptide. (B) Size-exclusion chromatography (SEC) profile of Nb1–Nb2 bivalent construct.
